# Single-Dose Radiation-Induced Oral Mucositis Mouse Model

**DOI:** 10.3389/fonc.2016.00154

**Published:** 2016-06-27

**Authors:** Osama Muhammad Maria, Alasdair Syme, Nicoletta Eliopoulos, Thierry Muanza

**Affiliations:** ^1^Experimental Medicine Department, Faculty of Medicine, McGill University, Montreal, QC, Canada; ^2^Radiation Oncology Department, Jewish General Hospital, McGill University, Montreal, QC, Canada; ^3^Lady Davis Institute for Medical Research, Jewish General Hospital, McGill University, Montreal, QC, Canada; ^4^Medical Physics Unit, McGill University, Montreal, QC, Canada; ^5^Oncology Department, McGill University, Montreal, QC, Canada; ^6^Surgery Department, Faculty of Medicine, McGill University, Montreal, QC, Canada

**Keywords:** epithelium, hydration, inflammation, mouse model, normal tissue injury, radiation-induced oral mucositis, radiation therapy, tongue

## Abstract

The generation of a self-resolved radiation-induced oral mucositis (RIOM) mouse model using the highest possibly tolerable single ionizing radiation (RT) dose was needed in order to study RIOM management solutions. We used 10-week-old male BALB/c mice with average weight of 23 g for model production. Mice were treated with an orthovoltage X-ray irradiator to induce the RIOM ulceration at the intermolar eminence of the animal tongue. General anesthesia was injected intraperitoneally for proper animal immobilization during the procedure. Ten days after irradiation, a single RT dose of 10, 15, 18, 20, and 25 Gy generated a RIOM ulcer at the intermolar eminence (posterior upper tongue surface) with mean ulcer floor (posterior epithelium) heights of 190, 150, 25, 10, and 10 μm, respectively, compared to 200 μm in non-irradiated animals. The mean RIOM ulcer size % of the total epithelialized upper surface of the animal tongue was RT dose dependent. At day 10, the ulcer size % was 2, 5, 27, and 31% for 15, 18, 20, and 25 Gy RT, respectively. The mean relative surface area of the total epithelialized upper surface of the tongue was RT dose dependent, since it was significantly decreased to 97, 95, 88, and 38% with 15, 18, 20, and 25 Gy doses, respectively, at day 10 after RT. Subcutaneous injection of 1 mL of 0.9% saline/6 h for 24 h yielded a 100% survival only with 18 Gy self-resolved RIOM, which had 5.6 ± 0.3 days ulcer duration. In conclusion, we have generated a 100% survival self-resolved single-dose RIOM male mouse model with long enough duration for application in RIOM management research. Oral mucositis ulceration was radiation dose dependent. Sufficient hydration of animals after radiation exposure significantly improved their survival.

## Introduction

Radiation-induced oral mucositis (RIOM) is a normal tissue injury side effect of ionizing radiation (RT) therapy with an 80% incidence in Head and Neck cancer patients (1, 2). In 2004, Scully et al. proposed four inflammatory stages during the clinical course of RIOM, which is considered a major dose-limiting toxicity (3, 4). RIOM clinical progress includes localized asymptomatic hyperemia and edema, then ulceration and confluent desquamation, then necrosis and possible secondary infection, then final fibrosis and/or repopulation (3). RIOM narrow therapeutic ratio leads to alteration in RT dose fractionation protocols, treatment interruptions, and poor local tumor control that can impact on long-term survival. Although considered a self-limited inflammation if the patient survives, RIOM could lead to a significant decline in quality of life in elderly sick patients, potentially necessitating alterations of the planned course of RT to lethal deterioration (1, 5, 6).

Generating a stable and well-characterized RIOM mouse model will facilitate current and future research studies aimed at repairing radiation-induced normal tissue injury. Some mouse models have been created in separate studies for both the fractionated (7–10) and the single-dose RT (1, 11–14). However, the short duration of such RIOM resulted in limitations in the experimental setup and performance. A study had recorded a fractionated-dose RIOM ulcer duration mean of 2.9 ± 0.7 days (8) (M ± SD) and a single-dose RIOM ulcer duration of 2.0 ± 0.4 days (M ± SD) (15). For this reason, we were interested in generating a RIOM mouse model with longer inflammatory and ulcerative phase duration that will allow for better experimentation and investigation of many injury variables within the same experiment, especially in translational research. This would lead to the generation of finer injury quantification and describing parameters to allow better RIOM injury control and therapy.

Our objective was to determine the highest single RT dose that will give a longer non-life threatening self-resolved RIOM in mice, with the longest possible inflammatory and ulcerative phase. Also, we aimed to histologically characterize such injury in order to precisely quantify the injury at that radiation dose for better treatment evaluation parameters.

## Materials and Methods

### Single-Dose RIOM Mouse Model

All animal handling was done according to McGill University’s Standard Operating Procedures (SOPs) and the Canadian Council of Animal Care (CCAC). The 8-week-old male BALB/c mice were purchased from Charles River® (Montreal, QC, Canada). Experiments were performed 2 weeks after the animal adjustment period (7 days) was completed at the animal facility. Generation of single-dose RIOM was done using Gulmay® orthovoltage X-ray D3225 irradiator (Suwanee, GA, USA) according to the following protocol. Average 25 g weighted 10-week-old male BALB/c mice were anesthetized with the Ketamine/Xylazine/Acepromazine anesthesia cocktail [prepared from 1 mL of ketamine (100 mg/mL), 0.5 mL xylazine (20 mg/mL), 0.3 mL acepromazine (10 mg/mL), and 8.2 mL of sterile isotonic saline or sterile water for injection]. We gave the anesthesia cocktail as 0.05–0.1 mL/10 g body weight, intraperitoneally, and protective ophthalmic ointment (natural tears) was applied (according to McGill University SOP-110). Afterward, mice were transferred to the radiation facility on electrical warming blankets. Mice were placed side-by-side in the prone along the borders of a 20 cm × 20 cm square cone (four or five animals per side) in a position that only allows the animal’s head to be irradiated (area from the mid-ear coronal plane until the tip of the nose was placed internally underneath the cone while the remaining animal body was outside the radiation field). We used an energy of 120 kVp and a central output of 115.8 cGy/100 monitor units (MU). Ionization chamber dosimetry was used to quantify the slightly reduced radiation dose near the periphery of the radiation field (where the mouse heads were located), and the number of MU was adjusted to compensate. We delivered 10, 15, 18, 20, and 25 Gy to induce RIOM. Animals were always kept on warm blankets to avoid hypothermia. After irradiation, animals were kept in a 33°C incubator for 2 h until complete recovery with subcutaneous hydration that continued for 24 h after RT (1 mL 0.9% saline subcutaneously/6 h for 24 h). Animals were moved to their cages with free access to enriched jelly food (Bio-Serv® Rodent Liquid Diet, AIN-76 served with 15% w/v gelatin) and water with daily observation. The primary and clinically relevant end-point was established as grade 3 RIOM by RTOG/EORTC scale version.2 (8, 15).

### Tissue Collection and Processing

At different time points (indicated for each experiment), animals were sacrificed and after cervical dislocation, complete tongue tissue and salivary gland were carefully dissected and placed immediately into cold 1× PBS. Tongues were stained with 1% toluidine blue (TB) in 10% acetic acid. Repeated wiping with acetic acid-soaked gauze was applied until no more dye could be recovered from the tissue (1). After macroscopical analysis of RIOM, tongue tissue was dissected longitudinally in the median plane (dividing the ulcer into identical halves), kept in 10% buffered formalin, and then paraffin embedded and 3 μm sections made for H&E staining and microscopic analysis. Many clinical and histological parameters for quantification of the RIOM were applied, e.g., we used ulcer size and ulcer size % (ImageJ® software measuring surface area in pixels was used for the ulcer size percentage of the total tongue epithelialized upper surface), ulcer duration, ulcer time-to-appear (latency), ulcer time-to-heal, posterior upper surface epithelium height (intermolar eminence epithelium height), and cellularity (infiltrating cells) as histological and clinically relevant parameters to quantify RIOM. In addition, we investigated the animal weight in relation to RIOM phase and severity.

### Statistics

GraphPad Prism® software (version 5.01) was applied for statistical analyses. Data were expressed as the M ± SEM. The paired two-tailed Student’s *t*-test was applied for two sets of data. *p*-Value <0.05 was considered a significant difference.

## Results

### RIOM Is a Radiation Dose-Dependent Injury

**Figure 1:**
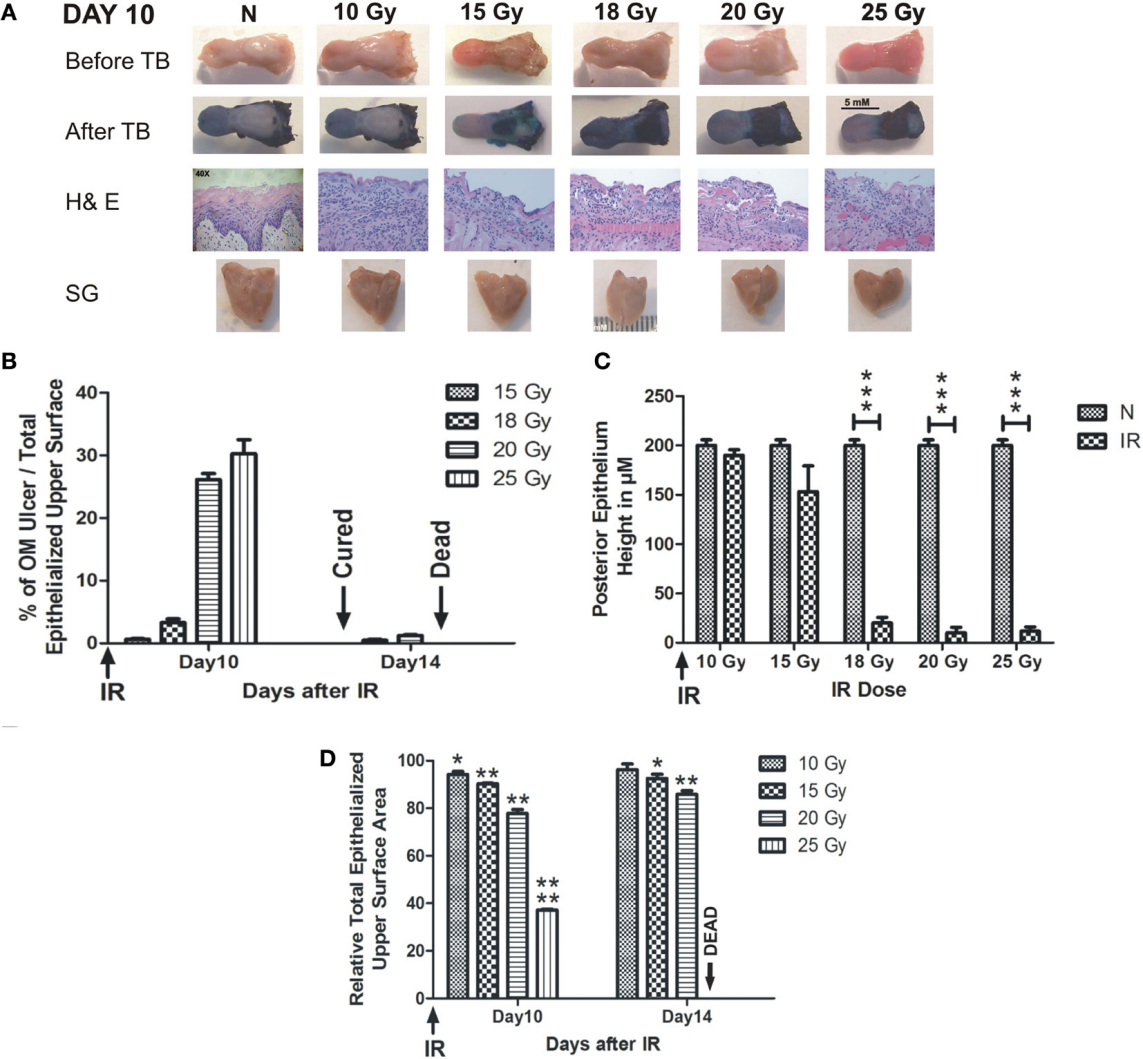
**RIOM is a radiation dose-dependent injury**. The 10-week-old mice were irradiated (IR) with a single dose of 10, 15, 18, 20, or 25 Gy at day 0 (four animals/dose). Animals were sacrificed at different time points, and tongues and salivary glands were collected. Tongues were stained with 1% TB in 10% acetic acid. After tongue imaging, the ulcer size and the total epithelialized upper surface of the tongue were measured in both non-irradiated (N) and irradiated animals at different RT doses. Then, tongues were kept in 10% buffered Formalin until the time of paraffin embedding. After embedding, 3-μm sections were stained with H&E. **(A)** Represents animals’ tongues before and after TB staining showing the posterior epithelium ulceration (in blue) at the intermolar eminence of the tongue, H&E staining, and salivary glands (SG) at day 10 after RT. **(B)** Represents the mean percentage of oral mucositis (OM) ulcer size of the total epithelialized upper surface of the tongue. **(C)** Represents the mean of the posterior epithelium height (ulcer floor within the intermolar epithelium) at different RT doses compared to non-irradiated animals (N) at day 10 irradiation. **(D)** Represents the mean relative total epithelialized upper surface area (relative = surface area in RT animal group/surface area in N animal group). Data from days 10 and 14 (*n* = 3) presented as the mean ± SEM. *p-value <0.05, **p-value <0.005, ***p-value <0.0005, and ****p-value <0.00005.

Radiation-induced oral mucositis ulceration was localized to the intermolar eminence of the animal tongue, which is located at the upper posterior surface of the tongue. All RT doses caused partial or complete loss of the eminence at day 7 after RT (Figure S1 in Supplementary Material). The TB-stained RIOM ulceration was evident with all RT doses except 10 Gy, which produced a very limited epithelial loss at the eminence; however, it showed marked cellular infiltration similar to other RT doses. All other RT doses showed complete loss of the eminence macroscopically. H&E staining showed complete loss of the eminence epithelium with RT doses of 15, 18, 20, and 25 Gy marked by the deep blue coloration of the TB. Ulceration was detectable at day 9 after RT in all experiments, while the eminence loss started at least 2 days earlier (Figure S1 in Supplementary Material). Collected salivary glands showed marked reduction in their total volume, with all RT doses compared to non-irradiated animals (Figure 1A). We found that the mean size percentage of RIOM ulcer relative to the total epithelialized upper surface of the tongue was RT dose dependent. More precisely, on day 10 after RT, it was 2, 5, 26, and 32% of the total epithelialized upper surface of the tongue for RT doses of 15, 18, 20, and 25 Gy, respectively. However, on day 14, the 15 Gy-generated ulcer was cured, and the 25 Gy-irradiated animals were dead due to extensive inflammation and dehydration, while the 18 and 20 Gy-generated ulcers were in the process of being repaired and still detectable by TB staining (Figure 1B).

We measured the RIOM ulcer floor epithelium height (eminence epithelium). We noted that although almost all epithelial layers were desquamated, there were still measurable micrometers of the epithelium (Figure 1A). We saw the marked increase in infiltrating cells at the sub-epithelial connective tissue as a sign of the inflammatory response. Minimal reduction in epithelium height was produced by 10 Gy, while it was more substantial than 15 Gy RT dose at day 10 after RT. However, the significant difference was documented only with 18, 20, and 25 Gy irradiation from day 9 to 13 (Figure 1C shows day 10 epithelium height in micrometers).

**Figure 2:**
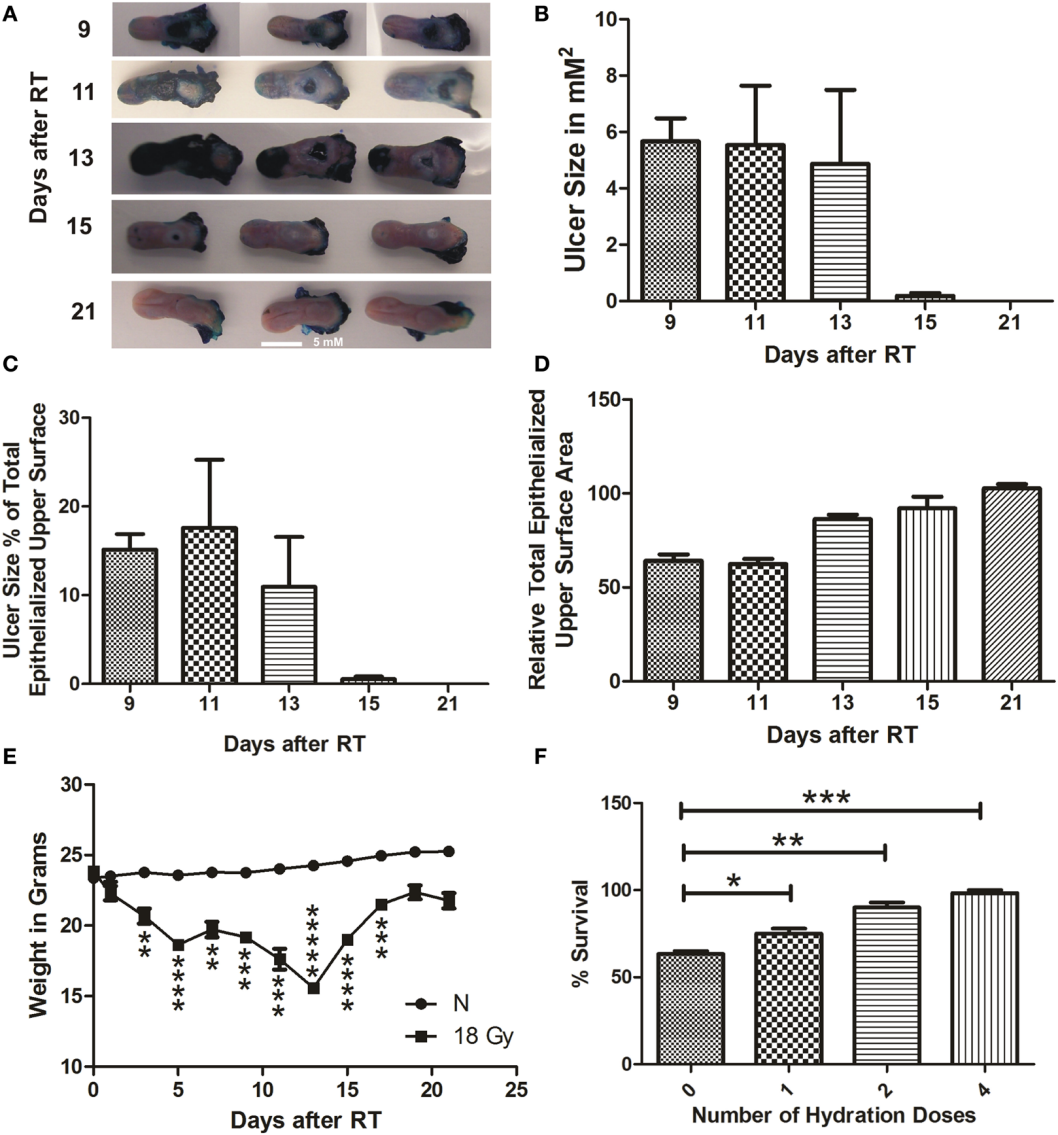
**RIOM model was established with a single RT dose of 18 Gy**. The 10-week-old mice were irradiated with a single dose of 18 Gy at day 0, 5 animals/time point. Animals were sacrificed at different time points and tongues were collected. Tongues were stained with 1% TB in 10% acetic acid. After tongue imaging, the ulcer size and the total epithelialized upper surface of the tongue were measured. We measured the animal weight every other day for 3 weeks after RT. **(A)** Represents the RIOM TB stained ulcers at the intermolar eminence of the tongue at different time points. **(B)** Represents the mean RIOM ulcer size in square millimeter **(C)** Represents the mean ulcer size percentage to the total epithelialized upper surface of the tongue. **(D)** Represents relative total epithelialized upper surface area. **(E)** Represents the mean animal weight for 3 weeks after RT. **(F)** Represents the percent survival of different hydration regimens used at the post-irradiation period. (*n* = 3), data presented as the M ± SEM. **p-value <0.005, ***p-value <0.0005, ****p-value <0.00005, and *****p-value <0.000005.

We measured the total epithelialized upper surface of the tongue in both non-irradiated and irradiated animals. We found a significant reduction in the total area of the epithelialized upper surface of the tongue with all irradiation doses (*p*-value <0.05–0.00005). In addition, we found that the mean of the relative total epithelialized upper surface area was RT dose dependent with all RT doses (relative = total epithelialized upper surface area of the tongue of irradiated animals/total epithelialized upper surface area of the tongue of non-irradiated animals). The mean of the relative total epithelialized upper surface was 97, 95, 89, 65, and 39% with 10, 15, 18, 20, and 25 Gy, respectively, at day 10 after RT. The epithelium surface area reduction reached maximum at days 9–11 after RT, then the surface area started to increase afterward to reach the normal range 3 weeks after RT. At day 14, animals irradiated with smaller doses started to recover (for example, 10 Gy-irradiated animals lost the significant surface area reduction at that time due to increased epithelial height), while animals irradiated with 25 Gy could not survive the severe inflammation (Figures 1D and 2D). We noted that, there was a significant improvement of animal survival when more hydration was provided during the post-irradiation period (Figure 2F).

### Self-Resolved Single-Dose RIOM with 100% Survival Rate

We were able to generate a self-resolved single-dose RIOM BALB/c male mouse model with 100% survival using the lowest possible RT dose, 18 Gy. We quantified the results as the M ± SEM. RIOM ulcer started to appear at day 9 (as evidenced by deep blue staining with TB) and resolved by day 15 in almost all experiments. With a single dose of 18 Gy, considering that the RT day is day 0, we achieved a self-resolved RIOM ulceration of 5.6 ± 0.3 days duration (95% confidence interval 4.233–7.1 days), ulcer time-to-appear (latency) at 9.3 ± 0.3 days (95% confidence interval 7.867–10.733 days), and ulcer time-to-heal at 15 ± 0.58 days (95% confidence interval 12.517–17.483 days). The RIOM ulcer was always at the posterior dorsal surface of the tongue where the intermolar eminence is located anatomically, called posterior epithelium or eminence epithelium (Figure 2A).

After 18 Gy RT, animals showed the largest ulcer mean size at days 9 and 11 after RT. Ulcer size was 5.7, 5.6, 5.0, 0.4, 0 μm^2^ at days 9, 11, 13, 15, 21, respectively (Figure 2B). The RIOM ulcer size percentage to the total epithelialized upper surface of the tongue was the highest at day 13 after RT. Ulcer size percentage was 16, 18.5, 12, 0.9, and 0% at days 9, 11, 13, 15, and 21, respectively.

Ulcer size was dependent on the stage of the RIOM, which resolved by 15 ± 0.58 days, nevertheless, the epithelium was completely recovered to normal mean heights at day 21 after RT (Figures 2A–C).

The relative total epithelialized upper surface of the tongue was RIOM stage dependent. The lowest surface area was recorded at days 9 and 11 after RT, which correspond to the largest recorded RIOM ulcer size. The mean of the relative total epithelialized upper surface was 60, 59, 78, 89, and 100% at days 9, 11, 13, 15, and 21, respectively (Figure 2D).

Animal weight loss and gain were significant parameters for the severity, the degree and the stage of the RIOM. After RT of 18 Gy, irradiated animals started to lose weight significantly (*p*-value <0.005), which reached the minimum by days 13 and 15 (*p*-value <0.000005 and 0.00005, respectively), which are the days following the largest ulcer size at days 9 and 11, then started to gain weight up to normal ranges by day 21 after RT (Figure 2E). In addition, we noted a RT-dependent reduction in salivary gland volume, and the total epithelialized upper surface of the tongue. This finding reflects the animal’s nutritional and hydration status.

We documented a significant improvement of the survival of RIOM animals in experiments with hydration doses of 1 mL of 0.9% saline subcutaneously/dose. This significant survival improvement was highest with four doses of hydration, one dose every 6 h for a total of 24 h after RT. The percentage survival was 63% with no hydration, and 72, 88, and 100% with 1, 2, and 4 doses, respectively, of hydration with 1 mL subcutaneous 0.9% saline/dose/6 h after RT (*p*-values <0.05, 0.005, and 0.0005, respectively for 1, 2, and 4 hydration doses) (Figure 2F).

## Discussion

We used grade 3 RIOM by RTOG/EORTC scale, Version.2, as our clinically relevant end point in generating our RIOM mouse model. Grade 3 signifies the presence of ulceration, extensive erythema, and inability to swallow hard food (4). As documented by Muanza et al. and Schmidt et al. (1, 15), we showed that RIOM severity is RT dose dependent. In addition to histological evidences of RIOM, macroscopical imaging showed earlier signs of intermolar eminence loss at least 48 h before the physical appearance of the ulcer on day 9. This finding was in agreement with what has been documented before using optical coherence tomography (1). The volume reduction noted in both tongue and salivary gland could be partially explained by the physical obstruction of food and fluid intake due to ulceration in addition to the systemic inflammatory reaction that disturbed the volume regulatory mechanism. We found a close correlation between the total epithelialized upper surface of the tongue and the animal weight. Forty-eight hours after the lowest total epithelialized upper surface had been seen, animal weight started to increase. Our results showed that proper hydration after RT is a critical life-saving procedure that is highly recommended for all post-irradiation care regimens.

We preferred using the ulcer size percentage to the total epithelialized upper surface of the tongue to express the severity and the stage of RIOM, in order to overcome the minimal individual variation in tongue size between animals of the same age. We were able to precisely identify the lowest single dose (18 Gy) delivered by orthovoltage RT to generate a self-resolved RIOM of 5.6 ± 0.3 days duration in BALB/c male mice. In addition, we were able to achieve a 100% survival rate after improving the post-irradiation hydration regimen. These two findings will significantly improve future studies on RIOM; mainly in translational research, where reliable clinically relevant therapeutic benefits are needed in a time-efficient manner. A mean RIOM ulcer duration of 5.6 days widened the tight ulcer duration recorded in earlier studies (10, 11).

To the best of our knowledge, this is the longest, and ever recorded single-dose RIOM ulcer duration in our mouse model. Such long RIOM ulcer duration will allow for better experimentation and investigation of many injury variables within the same experiment, especially in translational research. This may lead to the generation of finer injury quantification and describing parameters to allow better RIOM injury control and therapy.

## Conclusion

In RIOM, ulcer size, total upper epithelialized tongue surface, and intermolar epithelium height were radiation dose dependent. We generated a self-limited single-dose RIOM mouse model of 5.6 ± 0.3 days physical ulcer duration, 9.3 ± 0.3 days ulcer latency, 15 ± 0.56 ulcer time-to-heal, and 100% survival with 18 Gy orthovoltage radiation. Beyond 18 Gy of orthovoltage radiation, mice could not survive beyond 10 days due to severe mucositis that resulted in uncorrectable weight loss and dehydration. We recommend the use of animal weight loss as one important parameter for the severity and stage of the mucositis. We also recommend using the total tongue epithelialized upper surface as an indicator of mouse hydration status. More studies are needed to identify a parallel fractionated-dose mouse model with orthovoltage X-ray radiation. Our model achieved a longer physical ulcer duration with measurable tongue upper surface epithelium (ulcer floor). We found a relation between the injury severity and stage, and the mouse weight. Our model showed improved survival rates with better post-irradiation hydration regimens. All our findings may lead to improved future experimentation for better management of RIOM.

## Author Contributions

OM: conception and design, collection and/or assembly of data, data analysis and interpretation, manuscript writing, and final approval of manuscript. AS: conception and design. NE: conception and design, provision of study material, data analysis and interpretation, and final approval of manuscript. TM: conception and design, financial support, provision of study material, data analysis and interpretation, and final approval of manuscript.

## Conflict of Interest Statement

The authors declare that the research was conducted in the absence of any commercial or financial relationships that could be construed as a potential conflict of interest.

## Supplementary Material

Figure S1 **Lost intermolar eminence before physical ulcer appearance**. The 25 Gy-irradiated animals were sacrificed and tongue was dissected. Imaging was done before and after TB staining. Comparison was done between days 7, 9, and 10. Note the lost tongue eminence at day 7 with undetectable physical ulceration yet. Tongue shrinkage and volume reduction were always noted after irradiation (*n* = 3).
