# Supplementary figures and images for: Single-Dose Radiation-Induced Oral Mucositis Mouse Model
Source: Front Oncol. 2016 Jun 27;6:154. doi: 10.3389/fonc.2016.00154 (PMC4921469; doi:10.3389/fonc.2016.00154)

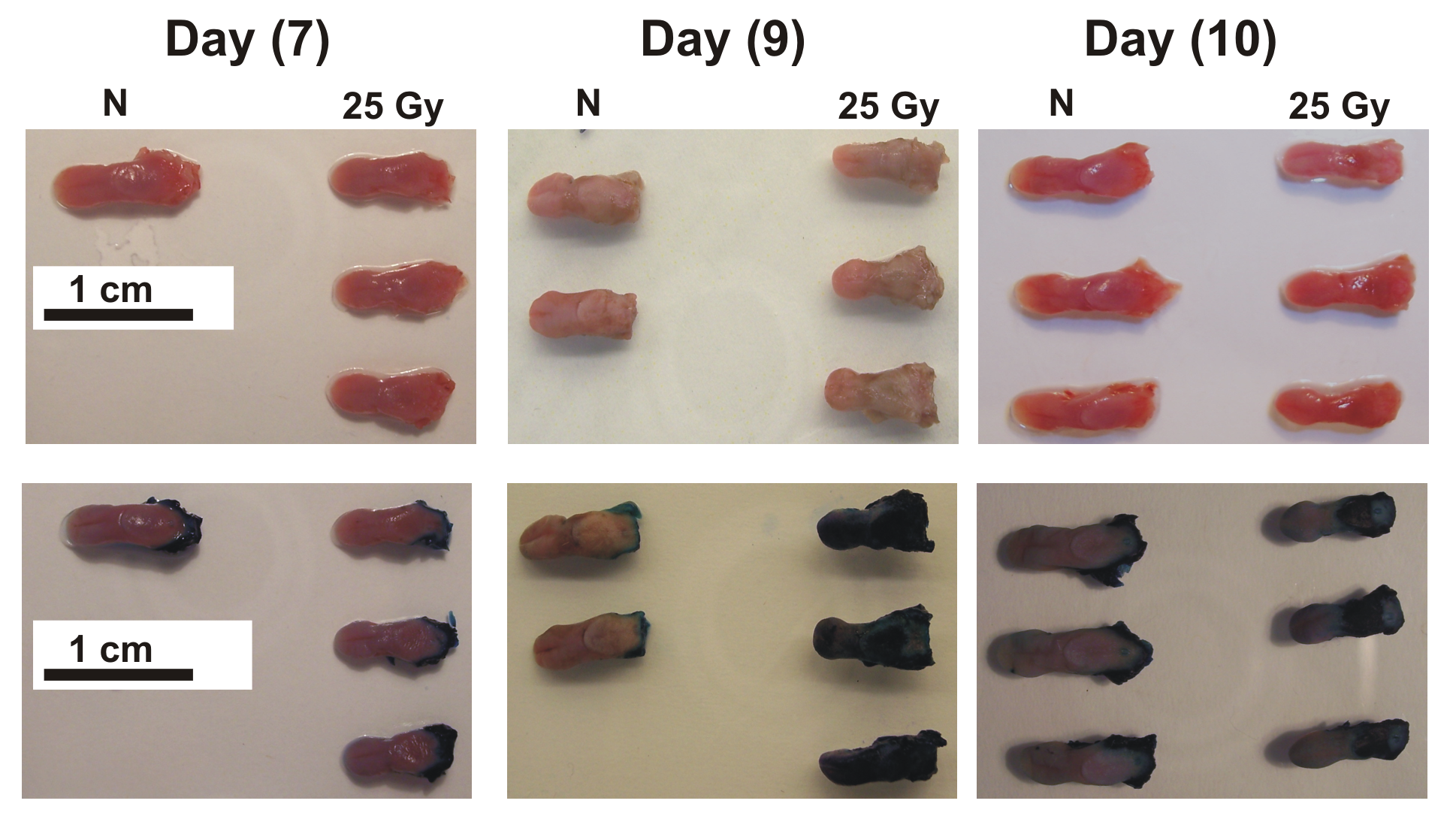

Supplement: Figure S1 — Lost intermolar eminence before physical ulcer appearance. The 25 Gy-irradiated animals were sacrificed and tongue was dissected. Imaging was done before and after TB staining. Comparison was done between days 7, 9, and 10. Note the lost tongue eminence at day 7 with undetectable physical ulceration yet. Tongue shrinkage and volume reduction were always noted after irradiation (n = 3). [file Image_1.tif]
